# Acid Tolerance of *Coxiella burnetii* Is Strain-Specific and Might Depend on Stomach Content

**DOI:** 10.3390/pathogens14030272

**Published:** 2025-03-12

**Authors:** Katharina Sobotta, Jan Schulze-Luehrmann, Martha Ölke, Katharina Boden, Anja Lührmann

**Affiliations:** 1 Institute of Medical Microbiology, Am Klinikum 1, 07747 Jena, Germany; 2 Mikrobiologisches Institut, Universitätsklinikum Erlangen, Friedrich-Alexander-Universität Erlangen-Nürnberg, 91054 Erlangen, Germany; 3 Synlab MVZ Weiden GmbH, MVZ Thuringia, Ernst-Ruska-Ring 15, 07745 Jena, Germany

**Keywords:** *Coxiella burnetii*, acid resistance, pepsin, milk

## Abstract

Q fever is a zoonotic disease caused by the obligate intracellular bacterium *Coxiella* (*C.*) *burnetii*. Human infections occur mainly via inhalation, but infections via the oral route have been observed. Gastric acidic conditions (pH 2–4) are the first defense mechanism to limit food-associated infections. In this study, we tested the ability of *C. burnetii* to survive extremely acidic conditions (pH 2–3) to assess the risk of oral infection in humans. We treated different *C. burnetii* strains with different pH values and calculated the recovery rate by counting colony-forming units. The analysis of an additional eight *C. burnetii* strains showed that some strains are acid-resistant, while others are not. Importantly, the presence of pepsin, an endopeptidase and the main digestive enzyme in the gastrointestinal tract, increases the survival rate of *C. burnetii*. Similarly, the presence of milk might also increase the survival rate. These results suggest that oral infections by *C. burnetii* are possible and depend on the bacterial strain and the stomach microenvironment. Consequently, the digestive infection route of *C. burnetii* could play a role in the transmission of the pathogen.

## 1. Introduction

*Coxiella* (*C.*) *burnetii*, a Gram-negative obligate intracellular bacterium, is the causative agent of Q fever, a widely distributed zooanthroponosis. After an incubation time of roughly two weeks, Q fever can manifest as an acute, self-limiting flu-like illness with complications such as pneumonia and hepatitis. Chronic disease occurs rarely and presents mainly as endocarditis or vasculitis [1]. The most common sources for *C. burnetii* transmission to humans are domestic ruminants. Humans become infected by aerosols derived from contaminated abortion material, birth products, urine, or feces. Asymptomatic, chronically infected cattle permanently shed *C. burnetii* in milk [2,3]. Consequently, the consumption of raw milk and dairy food products bears the potential risk of oral human infection.

Different studies could prove that dairy products (raw milk, yogurt, cheese) of unpasteurized milk contained viable *C. burnetii* [4,5,6,7]. Concentrations of *C. burnetii* in milk can reach more than 10^6^ bacteria in 250 mL [8]. For a study, volunteers consumed *C. burnetii*-contaminated raw milk. None of them developed any symptoms of disease [9]. Therefore, the oral transmission of *C. burnetii* is still controversially discussed. Experiments with mice infected by oral gavage resulted in clinical symptoms, a serological response, and bacterial dissemination [10]. Furthermore, *C. burnetii* directly delivered into the stomach resulted in colonization of the gastrointestinal tract [11]. Importantly, the oral route of infection is the least efficient one [11,12]. Nevertheless, it represents a possible mode of transmission.

In the environment, *C. burnetii* is highly resistant to stress factors such as temperature, osmotic pressure, and chemicals. Once inside a host cell, the bacteria replicate in a large lysosome-like parasitophorous vacuole called the *C. burnetii*-containing vacuole (CCV). A unique characteristic of the CCV is its acidic environment (pH 4.5 to 5.3). *C. burnetii* requires acidic conditions for the activation of their metabolism [13,14] and of the type IV secretion system, an essential virulence factor [15]. The stomach is the first line of defense against bacterial infections from food intake. Gastric acid has a pH of 1 to 3 and contains digestive enzymes, including pepsin, to digest proteins. Different bacterial species like enteric bacteria (e.g., *Shigella* spp., *E. coli*, and *Yersinia enterocolitica*) and *Helicobacter pylori* have developed strategies to survive the extremely acidic milieu in the stomach [16,17] and are therefore regarded as acid-resistant. Acid resistance is defined as the percentage of survival of the bacterial inoculum exposed to pH 2.5 for 2 h. Gorden et al. redefined survival rates for enteric bacteria after acid treatment: (i) bacteria with a survival rate ≥ 10% of the inoculum considered acid-resistant and (ii) bacteria with survival rates less than 0.001% of the inoculum as acid-sensitive [16]. The aim of this study was to analyze the survival rate of various *C. burnetii* strains under extremely acidic conditions simulating the human stomach environment to assess the risk of contracting Q fever by consuming contaminated food. Thus, we treated *C. burnetii* with various pH values for different time periods and calculated the recovery rate by determining the colony-forming units on ACCM-2 agar plates. In addition, we included pepsin, stomach medium, and milk in our experiments to determine their influence on *C. burnetii* survival.

## 2. Materials and Methods

Chemicals were purchased from Sigma Aldrich, Darmstadt, Germany unless stated otherwise.

***Coxiella burnetii* strains.** Nine different *C. burnetii* strains were selected for the acid survival experiments and are listed in Table 1. All strains were grown in acidified citrate cysteine medium-2 (ACCM-2) (Sunrise Science Products, San Diego, CA, USA) [18,19] for ten days at 37 °C, 2.5% O_2_, and 5 % CO_2_ prior to storage. Bacteria were harvested and stored in cell-freezing media (ACCM-2 with 10% glycerol) at −40 °C. All experiments with *C. burnetii* strains were performed in a Biosafety Level-3 (BSL-3) laboratory with BSL-3 standard operating procedures.

*C. burnetii* (Nine Mile RSA439 phase II, 1 × 10^6^/mL) was incubated in ACCM-2 medium at 37 °C, 2.5% O_2_, and 5% CO_2_ for five days. Bacteria were pelleted for 15 min at 4500× *g* and 4 °C and resuspended in PBS pH 7.4 to a concentration of 2 × 10^10^/mL after quantification by optical density at 600 nm (OD_600_), where an OD_600_ of 1 equals 1 × 10^9^ *C. burnetii* per mL. Aliquots were stored for up to six months at −80 °C and used directly for the experiments. Experiments using Nine Mile phase II were performed under BSL-2 conditions.

**2x stomach medium**: 4.8 g/L NaCl, 1.56 g/L NaHCO_3_, 2.2 g/L KCl, 0.22 g/L CaCl_2_; adjust pH to 2.5 with 5 M HCl and autoclave 20 min at 121 °C; store at 4 °C for up to one month.

Dissolve 13.5 g **formula** (Holle Bio Anfangsmilch 1 demeter; Holle baby food AG, Riehen, Germany) in 85 mL sterile distilled water (a.d.) and adjust the pH to 2.5 with 5 M HCl, fill up to 100 mL; store aliquots at −20 °C for up to six months.

A **pepsin stock solution** was prepared at 10 mg/mL in PBS, pH 2.5 or in 2x stomach medium. Aliquots were stored at −20 °C for up to six months.

**Determination of acid resistance.** Acid resistance evaluation of *C. burnetii* strains was carried out in PBS with pH values of 2.0 to 4.0 as well as pH 7.4 as control. The pH was adjusted to the desired value with 37% HCl (Carl Roth, Karlsruhe, Germany) and sterile-filtered. Duplicates of each 100 µL pH-conditioned PBS were mixed with 5 µL of 2 × 10^10^/mL *C. burnetii* in 1.5 mL reaction tubes. After incubation time at 37 °C and 5% CO_2_, samples were neutralized with 900 µL PBS (pH 7.4), and the viable bacteria amount was calculated by CFU determination (see below).

**Evaluation of proteolytic activity on the survival of *C. burnetii.*** The pepsin stock solution of 10 mg/mL was diluted with PBS (pH 2.5 or 7.4) to 2, 1, and 0.5 mg/mL. Duplicates of each 100 µL dilution were mixed with 5 µL of 2 × 10^10^/mL *C. burnetii* in 1.5 mL reaction tubes. After incubation for 2 h or 3 h at 37 °C and 5% CO_2_, samples were neutralized with 900 µL PBS (pH 7.4), and the viable bacteria were calculated by CFU determination (see below).

**Investigation of the role of formula on the survival of *C. burnetii* in an acidic environment.** The pepsin stock solution of 10 mg/mL was diluted with 2x stomach medium, pH 2.5 to 4 mg/mL. Equal volumes of 2× stomach medium with or without pepsin were premixed with a.d., pH 2.5 or with formula. Duplicates of each 100 µL dilution and, as controls, 100 µL PBS with pH 2.5 and pH 7.4 were combined with 5 µL of 2 × 10^10^/mL *C. burnetii* in 1.5 mL reaction tubes. After an incubation time of 2 h and 3 h at 37 °C and 5% CO_2_, samples were neutralized with 900 µL PBS (pH 7.4), and the viable bacteria were calculated by CFU determination (see below).

**Determination of colony-forming units (CFU).** 2x ACCM-2 medium was prepared as described by the manufacturer and adjusted to pH 4.75 with 6 N NaOH, sterile-filtered, and stored at 4 °C for up to 4 weeks. An equal volume of sterile 0.5% agarose in a.d. was mixed with 2x ACCM-2 medium, and 20 mL was used to pour a 10 cm plate. Serial 10-fold dilutions of the *C. burnetii* suspension in 1x ACCM-2 medium were prepared in a 96-well dish. Between each dilution step, the bacterial suspension was resuspended thoroughly at least 10 times. The CFU titer was determined by spotting 10 µL of each diluted bacterial suspension on the top of the agarose plate. Three spots were spotted on the plate per dilution and served as technical replicates. ACCM-2 agarose plates were incubated for 9 to 14 d at 37 °C, 2.5% O_2_, and 5% CO_2_. Thereafter, colonies were counted, and the concentration of viable bacteria (CFU/mL) was calculated.

**Re-infection of buffalo green monkey (BGM) cells.** A total of 6 × 10^4^ BGM cells were seeded into 24-well culture plates (tissue-culture treated, Greiner bio-one, Frickenhausen, Germany) and cultured for two days until they reached 80% confluence. *C. burnetii* were incubated for 2 h in PBS adjusted to different pH values. Cells were infected with the pH-conditioned bacteria for 24 h at 37 °C and 5 % CO_2_. Infected cells were washed with pre-warmed PBS (37 °C), and BGM medium (OptiMEM, 5% FCS) was added for further cultivation. After incubation times of 1 and 14 d, the cells were sampled by washing the BGM cell layer three times with pre-warmed PBS and detaching by treatment with 100 µL trypsin/EDTA. After a 10 to 15 min incubation at 37 °C, the trypsin was neutralized with 400 µL PBS. The samples were then stored at −40 °C. For determination of the bacteria concentration, the samples were treated with three freeze (−40 °C) and thaw cycles to release the bacteria from their vacuoles. For PCR detection, 100 µL aliquots of the samples were inactivated by boiling for 20 min at 95 °C. The number of genome equivalents (GEs) was monitored by quantification of the isocitrate dehydrogenase (*icd*) gene by quantitative real-time PCR (qPCR) [20]. A replication factor was calculated by dividing the log10 values of GEs detected at day 14 post-infection by the respective values detected at day 1 post-infection.

**Immunofluorescence.** EA.hy926 cells were seeded on coverslips in a 24-well plate and incubated overnight. Cells were infected with *C. burnetii* with MOI 100. At 48 h post-infection, the cells were fixed with 4% paraformaldehyde (Alfa Aesar, Haverhill, MA, USA) in PBS (Biochrom GmbH, Berlin, Germany) for 15 min at room temperature in the dark, permeabilized with ice-cold methanol for 1 min, and quenched with 50 mM NH_4_Cl (Roth) in blocking buffer (PBS with 5% goat serum; Thermo Fisher Scientific, Darmstadt, Germany) for 30 min at room temperature. The coverslips were incubated with anti-LAMP2 (H4B4, DSHB) and anti-*C. burnetii* antibodies [21] in blocking buffer, washed 3 times with PBS, and incubated with the secondary Alexa Fluor-labeled antibodies Alexa 488 and Alexa 594 (Dianova, Hamburg, Germany) in blocking buffer. The cells were mounted using ProLong Diamond with DAPI (Thermo Fisher Scientific, Darmstadt, Germany). Analysis was performed using a Carl Zeiss LSM 700 Laser Scanning Confocal Microscope, recording data with a 63x/1.4 oil immersion objective lens and Zen software (Zen 3.0 SR (black) 16.0.1.306) (Carl Zeiss, Oberkochen, Germany).

**Statistical analysis.** Statistical comparisons were conducted using a *t*-test with statistic software SPSS (version 24), and a significant difference was established as *p* ≤ 0.01.

## 3. Results

**Figure 1:**
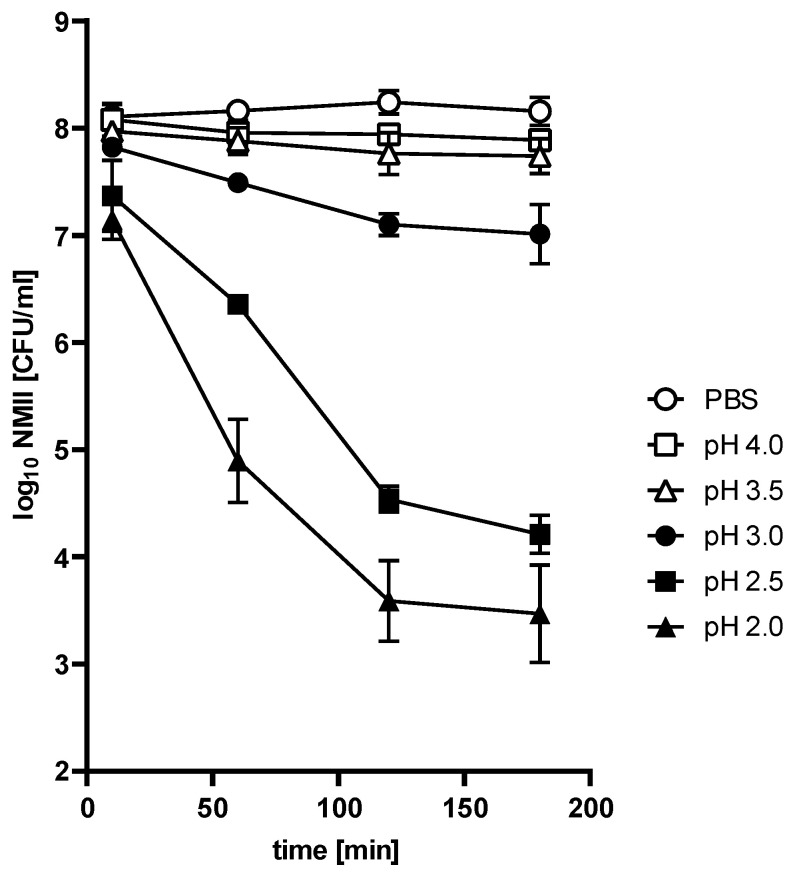
**Acid resistance of stationary phase *C. burnetii* NMII.** NMII (1 × 10^8^ CFU/mL) were incubated in different pH values (pH 2–4) for 10, 60, 120, and 180 min. PBS (pH 7.3) served as a control. The recovery rate of NMII was determined by CFU calculation on ACCM-2 agar plates. CFU values (log_10_ CFU) represent the mean of three independent experiments, each with three technical replicates and standard errors for six samples.

**The *C. burnetii* NMII strain is acid-resistant.** To determine whether *C. burnetii* would be able to survive the stomach passage, we treated stationary phase *C. burnetii* NMII (10 days in ACCM-2) [22] with different pH values for different time periods. As a control, we incubated NMII in PBS at pH 7.4. Pathogen survival was evaluated by the ability to grow on ACCM-2 plates. As shown in Figure 1, the survival of NMII depended on incubation time and pH value. In comparison to the PBS control, hardly any change in the bacterial recovery rate was observed after treatment at pH 3.5 and 4 over a period of 180 min. However, treatment with pH 2, 2.5, and 3 reduced bacterial viability. The recovery rate of live bacteria decreased exponentially by 0.5 log at pH 3 to 4.3 log at pH 2 after 120 min incubation. There was no further reduction for the remainder of the experiment under all tested conditions. Thus, acid tolerance was observed at all pH values. Importantly, the percent survival rate of NMII after 120 min at pH 2.5 was 0.02%. According to the definition [16], NMII belongs to the acid-resistant group.

**Figure 2:**
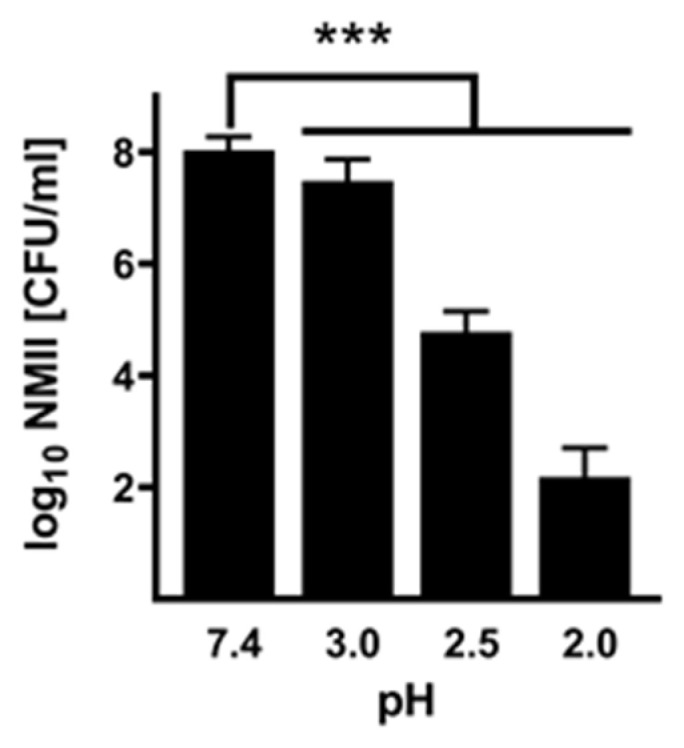
**Acid resistance of exponentially growing *C. burnetii* NMII.** NMII (1 × 10^8^ CFU/mL) were incubated in different pH values (pH 2–3) for 3 h. PBS (pH 7.4) served as a control. The recovery rate of NMII was determined by CFU calculation on ACCM-2 agar plates. CFU values (log_10_ CFU) represent means and standard errors of four independent experiments, each with two biological and three technical replicates. *** *p* < 0.001.

Bacteria might have different physiological properties depending on their growth phase. The experiments presented in Figure 1 were performed with stationary-phase bacteria. To exclude an influence of the growth phase on acid resistance, we incubated exponentially growing bacteria for 3 h in PBS of different pH values. The growth phase did not seem to affect acid resistance (Figure 2). Thus, pH 3 only slightly influenced the viability of exponential-phase bacteria. In contrast, pH 2.5 and pH 2 strongly reduced bacterial viability. Similar results were obtained with stationary-phase bacteria (Figure 1).

**Figure 3:**
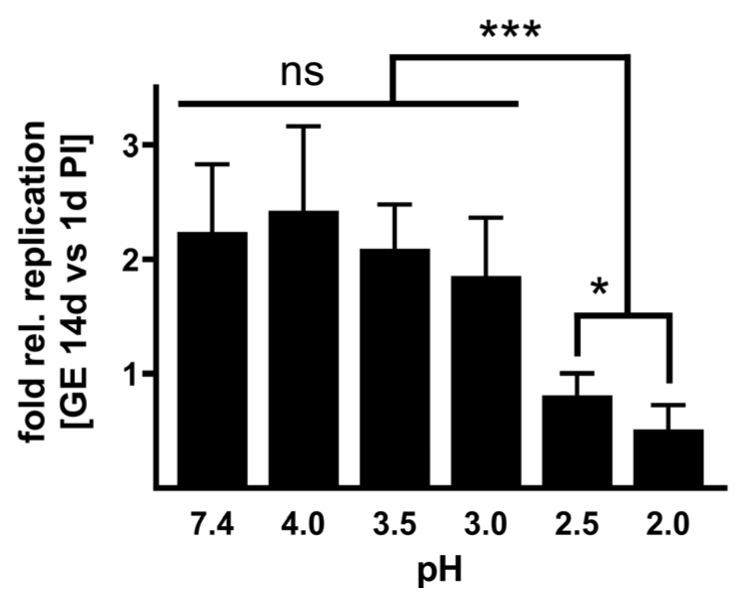
**Infectivity of acid-treated *C. burnetii* NMII.** BGM cells were infected with NMII pre-treated with different pH values (pH 2–4). NMII incubated in PBS (pH 7.4) served as a control. The number of GEs was quantified at 1 and 14 days post-infection. The increase in bacterial genomes was calculated as a replication factor representing the ratio between 1 and 14 days post-infection. The mean values and standard deviations of three different experiments are shown. ns > 0.05, * *p* < 0.05, *** *p* < 0.001.

**Infectivity of acid-treated NMII.** To investigate whether the acid treatment influences the infectivity of *C. burnetii*, we infected BGM cells with NMII, which were pre-treated for 2 h with different pH values or PBS as control. After 1 and 14 days, we purified the DNA of the samples and determined the *C. burnetii* genomic equivalents (GEs). The ability to replicate was calculated by a replication factor of *C. burnetii* (calculated by dividing log10 values of GE at day 14 by the value detected at day 1 post-infection). *C. burnetii* treated with a weakly acidic solution of pH 3 to 4 retained their ability to replicate intracellularly (Figure 3). In contrast, *C. burnetii* treated with strong acidic concentrations of pH 2 to 2.5 showed no or only minor replication. These data stress that a pH of 2 or 2.5 reduces *C. burnetii* viability (Figure 1) and, consequently, the ability to replicate intracellularly (Figure 3).

**Figure 4:**
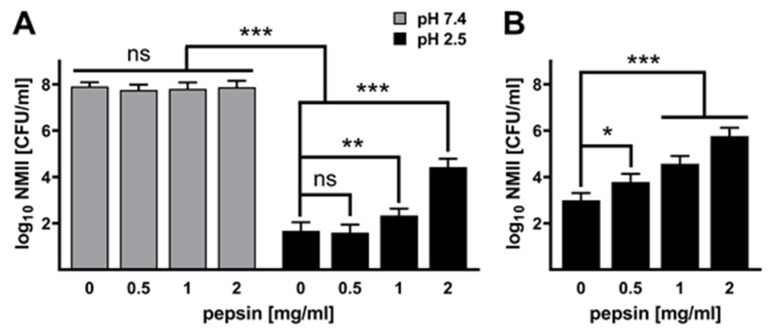
**Influence of pepsin on the acid resistance of *C. burnetii* NMII.** NMII (1 × 10^8^ CFU/mL) were treated with the pepsin concentrations indicated at pH 2.5 for 2 h (**A**) and for 3 h (**B**). As a control, NMII were cultured in PBS at pH 7.4 for 2 h (**A**) with the pepsin concentrations indicated. The recovery rate of NMII was determined by CFU calculation on ACCM-2 agar plates. CFU values (log_10_ CFU) represent the means and standard errors of four independent experiments, each with two biological and three technical replicates. ns > 0.05, * *p* < 0.05, ** *p* < 0.01, *** *p* < 0.001.

**Pepsin influences the acid tolerance of *C. burnetii.*** Pepsin is the main digestive enzyme in gastric juice. It is activated from its precursor pepsinogen by the stomach acid. Pepsinogen itself is produced by gastric chief cells in the stomach wall. We analyzed the impact of this enzyme on the acid tolerance of *C. burnetii*. Thus, we treated *C. burnetii* with different concentrations of pepsin at pH 7.4 or pH 2.5 for 2 h. The addition of pepsin at neutral pH to axenically grown *C. burnetii* did not influence the viability of the pathogen (Figure 4A). In contrast, at pH 2.5, pepsin increased the acid tolerance of *C. burnetii* in a dose-dependent manner (Figure 4A). Comparable results were obtained when we treated *C. burnetii* at pH 2.5 and different concentrations of pepsin for 3 h (Figure 4B). Thus, the presence of pepsin increased the acid tolerance of *C. burnetii*.

**Figure 5:**
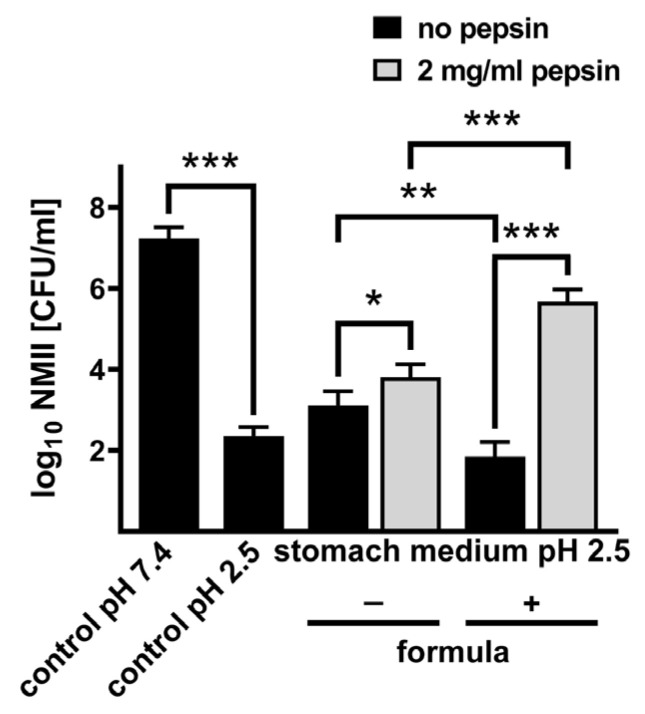
**Influence of formula in combination with pepsin on survival of *C. burnetii* NMII.** NMII (1 × 10^8^ CFU/mL) were incubated for 2 h with stomach medium at pH 2.5 with or without 2 mg/mL pepsin. In addition, formula was either added or not. As controls, PBS at pH 2.5 and pH 7.4 was used. The recovery rate of NMII was determined by CFU calculation on ACCM-2 agar plates. CFU values (log_10_ CFU) represent the means and standard errors of four independent experiments, each with two biological and three technical replicates. * *p* < 0.05, ** *p* < 0.01, *** *p* < 0.001.

**Formula in combination with pepsin boosts acid tolerance and infectivity of *C. burnetii.*** Bacterial survival upon passage through the stomach is influenced not only by the pH and the presence of pepsin but also by food and stomach medium, which contains several ions. To mimic conditions in the human stomach, we used an artificial stomach medium [23]. To test the influence of milk and its components on *C. burnetii* acid tolerance, we used baby formula. First, we confirmed that pepsin at a concentration of 2 mg/mL increases *C. burnetii* viability not only in PBS (Figure 4A,B) but also in stomach media (Figure 5). Next, we aimed to analyze whether the addition of formula influences the acid tolerance of *C. burnetii*. However, the addition of formula to the medium increases the pH value, which consequently would allow the increased survival of *C. burnetii* (Figure 1 and Figure 2). Thus, we adjusted the pH value after the addition of the formula back to pH 2.5. We did not observe an increase in acidic tolerance if *C. burnetii* was incubated in stomach medium containing formula. Importantly, in the presence of stomach medium, pepsin, and formula, we observed an increase in survival of nearly 4 logs (Figure 5). These data suggest that pepsin might digest the formula into different products (amino acids), which might affect the acid tolerance of *C. burnetii*.

**Figure 6:**
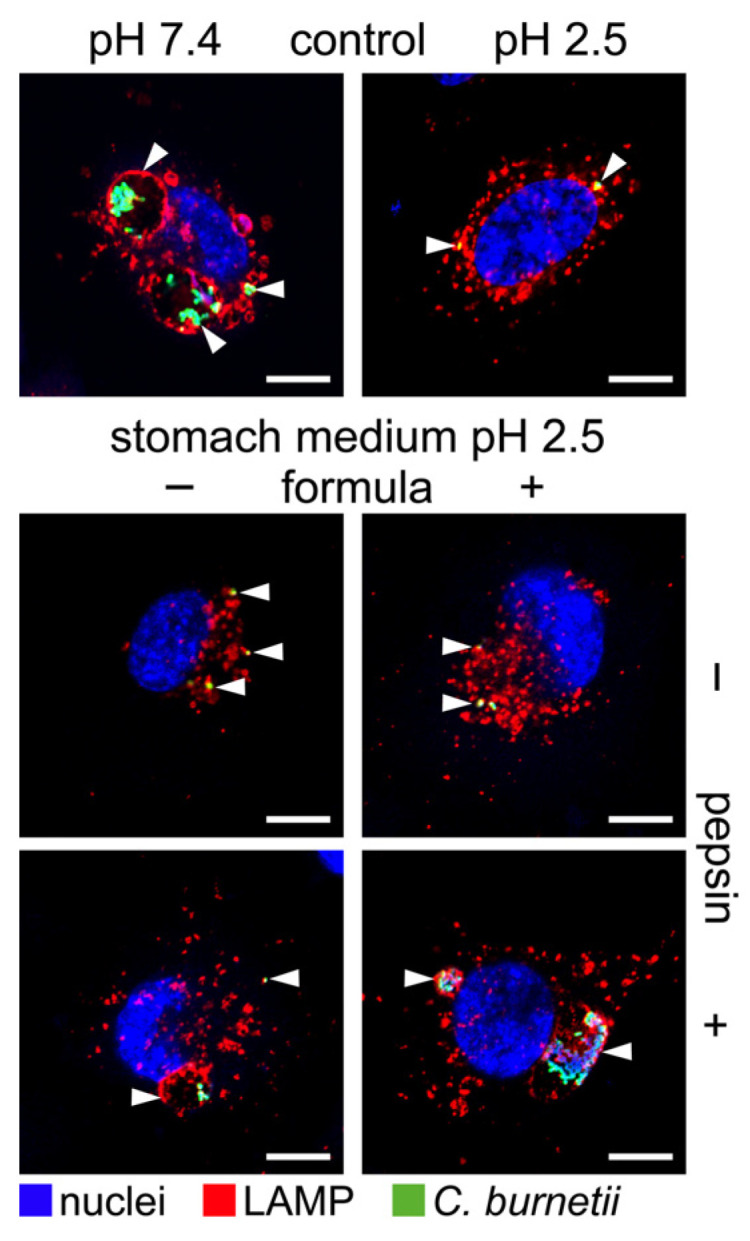
**Influence of a simulated stomach passage on the infectivity of the *C. burnetii* NMII.** Bacteria treated with either pepsin (20 mg/mL) and formula or not were used to infect endothelial EA.hy926 cells on coverslips with MOI 100. As controls, bacteria treated with PBS at pH 2.5 or pH 7.4 were used to infect EA.hy926 cells at MOI 100. The cells were fixed 48 h post-infection and stained for LAMP-1 (red), *C. burnetii* (green), and DNA (blue). Representative images are shown from three independent experiments. Arrows indicate *C. burnetii*-containing vacuoles. Each scale bar represents 10 μM.

Importantly, the treatment of *C. burnetii* in stomach medium with pepsin and formula at pH 2.5 allowed the pathogen to infect and establish a replicative CCV in endothelial cells, similar to bacteria incubated in PBS at pH 7.4 (Figure 6). In contrast, the incubation of *C. burnetii* in PBS at pH 2.5 or in stomach medium +/− pepsin at pH 2.5 prevented the ability of *C. burnetii* to replicate intracellularly (Figure 6). Taken together, the composition of the stomach content seems to be decisive for the ability of *C. burnetii* to survive the stomach passage as an infective agent.

**Figure 7:**
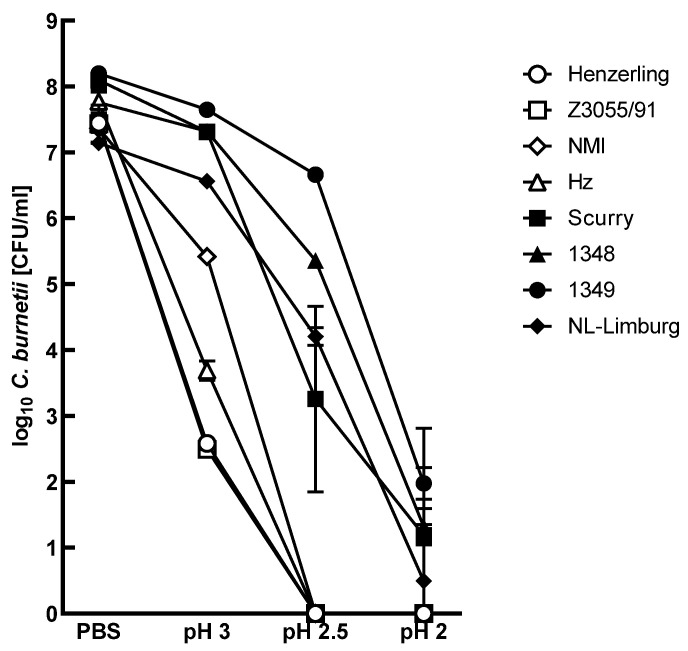
**Acid resistance of *C. burnetii* strains.** Different *C. burnetii* strains (1 × 10^8^ CFU/mL, Table 1) were incubated with pH 2, 2.5, and 3 for 120 min. PBS (pH 7.3) served as a control. The recovery rate was determined by CFU calculation on ACCM-2 agar plates. CFU values (log_10_ CFU) represent the means of 3 independent experiments, each with three technical replicates.

**Different *C. burnetii* strains show different acid tolerance.** So far, we used the model strain NMII to investigate the acid tolerance of *C. burnetii*. However, different *C. burnetii* isolates seem to have different activity profiles and physiological properties, as demonstrated by the ability to grow in axenic media [24] or by containing different subsets of virulence factors [25]. Therefore, we analyzed the acid tolerance of *C. burnetii* strains from different sources. Importantly, the response to the acid treatment was strain-specific (Figure 7). Indeed, acid treatment divided the *C. burnetii* strains into two groups: (I) acid-tolerant strains (survival rate between 0.001–10%) and (II) acid-sensitive strains (survival rate < 0.001%). The acid-tolerant strains, such as the human strains 1349, NL-Limburg, and Scurry, as well as the sheep strain 1348, were decreased in their viability by 1 to 3 log level at pH 2.5 with a survival rate between 0.01% (Scurry) and 2.86% (1349). Treatment with pH 2 reduced the live bacteria concentration of the acid-tolerant strains clearly and resulted in a survival rate lower than 500 bacteria. However, acid-sensitive strains, such as the human strains Henzerling and D-Jena, as well as the sheep strain Z3055/91 and model strain NMI, did not survive treatment with pH 2.5. Taken together, the acid tolerance of *C. burnetii* is strain-specific.

## 4. Discussion

Chronically infected ruminants, especially cattle, permanently shed *C. burnetii* in milk [26]. Nevertheless, consuming unpasteurized dairy products is considered to pose a low risk for Q fever infection in humans even though there is evidence that most bulk tank milk samples contain viable *C. burnetii* and that human consumption of *C. burnetii*-contaminated dairy products leads to a higher risk for seroconversion [27]. There are two possible ways of human infection by contaminated milk products: (i) By regurgitation of gastric content and aspiration into the respiratory tract. Several pulmonary syndromes may occur after aspiration, including aspiration pneumonia, which is defined as inhalation of oropharyngeal secretions that are colonized by pathogenic bacteria [28]. Especially, infants and elderly persons possess a higher risk. Indeed, milk aspiration might cause death in infants [29]; and (ii) by infection via the gastric route.

One early border of gastric host defense is the very low pH value of the stomach. The amount of pathogens that survive the gastric environment is influenced by stomach acidity. In this study, we analyzed the survival rate of *C. burnetii* under the extreme acidic condition of the human stomach. To determine the acid resistance of *C. burnetii,* we treated the reference strain NMII with pH values between 2.0 and 4.0 for different periods of time. The survival of NMII was dependent on incubation time and pH value (Figure 1 and Figure 2). With increasing treatment time, the recovery rate of NMII decreased for highly acidic conditions (pH < 3.0). In contrast, less acidic conditions (pH > 3.5) hardly affected bacterial vitality (recovery rate after 120 min: 35% of the inoculum). Under extremely acidic conditions (120 min, pH 2.5), NMII only survives with a recovery rate of 0.02%. According to the definition of Gorden and colleagues [16], NMII is classified as an acid-resistant bacterium. In contrast, gastrointestinal tract pathogens like *Shigella* spp. and *E. coli* survive under the same conditions with clearly higher rates (50 to 55%) [16]. These differences might be caused by the different main infection route of gastrointestinal bacteria versus respiratory pathogens, like *C. burnetii*, and, thus, different infection and adaptation strategies.

Several bacteria have evolved resistance mechanisms against the lethal pH value ≤ 2.5 by acid tolerance response (e.g., gas system, biofilm formation, proton pumps) to sense and adapt to an acidified environment [30]. Bacteria causing foodborne diseases need approximately 90 to 120 min after exposure to the triggering pH to mount a fully active acid response [31]. In our study, NMII develops a pH value-independent acid tolerance response between 120 and 180 min post-exposure (Figure 1). The mechanism responsible for the acid tolerance response in *C. burnetii* is currently unknown. However, this tolerance is not only important for the adaptation to the gastric environment because *C. burnetii* localizes in an acidic, lysosomal-like compartment within its host cells [32,33]. This obligate intracellular pathogen not only survives this harsh environment—it even requires it for intracellular replication [18]. Key to the ability to replicate is the presence of the type IVB secretion system (T4SS) [34], which is activated by the low pH within the CCV [15].

Furthermore, the acid treatment did not affect the ability of *C. burnetii* to invade eukaryotic cells (Figure 3 and Figure 6). However, it might be possible that passage through the acidic stomach might limit the ability of *C. burnetii* to replicate intracellularly (Figure 3 and Figure 6). In the future, it will be interesting to investigate the invasion and replication of *C. burnetii* in human colon cells after acid treatment to assess the risk for intestinal entry of *C. burnetii* in humans. In cattle, intestinal epithelial cells permit the invasion of *C. burnetii* and serve as a possible entry site for dissemination in the body but not as host cells for bacterial replication [35].

Different clinical outcomes of Q fever in humans and animals have been associated with distinct virulence in genomic groups of *C. burnetii* [36,37]. In this study, we investigated the acid stress reaction of different *C. burnetii* strains (Figure 7). The recovery rate of *C. burnetii* showed considerable differences between the strains. They could be divided into two groups: acid-resistant (1349, NL-Limburg, Scurry, 1348) and acid-sensitive strains (Henzerling, D-Jena, Z3055/91, NMI). The acid response of the strains was independent of the strain’s original host (human, sheep, tick) and the genotype. *C. burnetii* strains isolated from humans (Henzerling, Hz, Scurry, 1349, NL-Limburg), sheep (Z3055/91, 1348), or ticks (NM) reacted differently to pH 2.5 treatment. The Dutch outbreak strain NL-Limburg survived the acid treatment (pH 2.5, 120 min) with a recovery rate of 0.12 %. NL-Limburg belongs to the same genotype (MST33) as the sheep strain Z3055/91, and a genome comparison indicated a high agreement with the genome of strain Henzerling (MST18) [38]. In our study, both Z3055/91 and Henzerling did not survive the acid treatment. The genome comparison found mutations in genes coding for ankyrin repeat domain proteins, membrane proteins, and proteins involved in translation and transcription [39]. These mutations could influence the acid stress response of NL-Limburg. Furthermore, the acid response of *C. burnetii* showed differences with respect to the LPS phase type. Virulent NMI, with a full-length LPS, did not survive a pH 2.5 treatment for 120 min. In contrast, avirulent NMII, which only lacks LPS O-polysaccharide chains [40], survived the treatment. These findings are contrary to the decreasing acid resistance of enterobacteria that lack LPS O antigens [41,42]. In the future, the strain-specific mechanism(s) responsible for the acid response under extremely acidic conditions should be identified and characterized to assess the risk for the oral transmission of *C. burnetii* strains.

The hypothesis that the consumption of *C. burnetii*-contaminated dairy products can lead to foodborne Q fever in humans is controversial. Our data suggest that the composition of the stomach content might be decisive for *C. burnetii* acid tolerance and, thus, its ability to survive passage through the stomach in a physiological state that retains pathogen infectivity. Thus, the presence of pepsin and formula allows the survival of *C. burnetii* even at pH 2.5, which, consequently, sustains the infectivity and ability of the bacteria to replicate intracellularly (Figure 4, Figure 5 and Figure 6). Especially, the combination of pepsin and formula alters the acid tolerance of *C. burnetii*, suggesting that individual milk components liberated by pepsin might be the trigger (Figure 5 and Figure 6). Milk and milk components have already been described for several bacteria as being protective during the stomach passage. Thus, the survival of *Bacillus cereus* strains was improved by milk products [43], and the viability of *Bifidobacteria* was increased by milk components during a simulated stomach passage [44]. In general, food is a protective factor for bacteria during passage through the stomach, as it increases the stomach pH. However, in addition to the buffering function of food, milk components might provide further signals, allowing the bacteria to adapt to the low pH within the stomach.

One risk for oral transmission is the fluctuating acid concentration in the human stomach. The pH value of adult gastric juice increased depending on the food composition, from pH 2.0 to pH 5, and returned to a normal value within an hour [45]. In contrast, the pH value of newborn infants increased from pH 3.5 to pH 6.4 after feeding and dropped to a normal value after 210 min [46]. The results of the study suggest that depending on bacterial strain, pH value, stomach content, and time, *C. burnetii* can survive passage through the stomach. As a consequence, persons with impaired acid production by functional dyspepsia and infants seem to have an enhanced risk for *C. burnetii* survival after passing the stomach. The Q fever guidelines (RKI, CDC) recommend that (I) *C. burnetii*-infected women should not feed their newborns by breastfeeding and (II) persons at particular risk should avoid the consumption of unpasteurized milk products. In the future, further studies are needed to determine the risk of food transmission for *C. burnetii* and the role of the digestive infection route in the manifestation of Q fever in humans.

## Figures and Tables

**Table 1:** *Coxiella burnetii* strains used in this study.

*C. burnetii* Strain	Infection of Host		Immediate Host	Source
	Disease	Course		
Nine Mile I (493)	N/A	acute	tick	1
Nine Mile II (439)	N/A	N/A	tick	1
Henzerling	pneumonia	acute	human	2
D-Jena	endocarditis	chronic	human	3
Scurry	hepatitis	chronic	human	1
Z3055/91	abortion	N/A	sheep	1
15QC1348	abortion	chronic	sheep	4
15QC1349	N/A	N/A	human	4
NL-Limburg	Aortic aneurysm	chronic	human	4

N/A = not available. 1. Friedrich-Loeffler-Institute; Institute of Molecular Pathogenesis, Jena. 2. Institut für Mikrobiologie der Bundeswehr, München. 3. Institute of Microbiology, Friedrich-Schiller-Universität Jena. 4. Friedrich-Loeffler-Institute, National Reference Laboratory for Q fever, Jena.

## Data Availability

The original contributions presented in this study are included in the article. Further inquiries can be directed to the corresponding author.
